# Field Application of SD Bioline Malaria Ag Pf/Pan Rapid Diagnostic Test for Malaria in Greece

**DOI:** 10.1371/journal.pone.0120367

**Published:** 2015-03-24

**Authors:** Maria Tseroni, Danai Pervanidou, Persefoni Tserkezou, George Rachiotis, Ourania Pinaka, Agoritsa Baka, Theano Georgakopoulou, Annita Vakali, Martha Dionysopoulou, Irene Terzaki, Andriani Marka, Marios Detsis, Zafiroula Evlampidou, Anastasia Mpimpa, Evdokia Vassalou, Sotirios Tsiodras, Athanasios Tsakris, Jenny Kremastinou, Christos Hadjichristodoulou, MALWEST Project

**Affiliations:** 1 Department of Hygiene and Epidemiology, Faculty of Medicine, University of Thessaly, Larissa, Greece; 2 Hellenic Centre for Disease Control & Prevention (KEELPNO), Athens, Greece; 3 General Hospital of Sparti, Lakonia, Sparti, Greece; 4 Médecins Sans Frontières, Athens, Greece; 5 Department of Parasitology, Entomology and Tropical Diseases, National School of Public Health, Athens, Greece; 6 Department of Microbiology, Faculty of Medicine, National and Kapodistrian University of Athens, Athens, Greece; Université Pierre et Marie Curie, FRANCE

## Abstract

Greece, a malaria-free country since 1974, has experienced re-emergence of *Plasmodium vivax* autochthonous malaria cases in some agriculture areas over the last three years. In early 2012, an integrated control programme (MALWEST Project) was launched in order to prevent re-establishment of the disease. In the context of this project, the rapid diagnostic tests (RDT) of SD Bioline Malaria Ag Pf/Pan that detects hrp-2 and pan-LDH antigens were used. The aim of this study was to assess the field application of the RDT for the *P*. *vivax* diagnosis in comparison to light microscopy and polymerase chain reaction (PCR). A total of 955 samples were tested with all three diagnostic tools. Agreement of RDT against microscopy and PCR for the diagnosis of *P*. *vivax* was satisfactory (K value: 0.849 and 0.976, respectively). The sensitivity, specificity and positive predictive value of RDT against PCR was 95.6% (95% C.I.: 84.8-99.3), 100% (95% C.I.: 99.6-100.0) and 100% (95% CI: 91.7-100.0) respectively, while the sensitivity, specificity and positive predictive value of RDT against microscopic examination was 97.4% (95% C.I.: 86.1-99.6), 99.4% (95% C.I.: 98.6-99.8) and 86.1% (95% CI: 72.1-94.7), respectively. Our results indicate that RDT performed satisfactory in a non-endemic country and therefore is recommended for malaria diagnosis, especially in areas where health professionals lack experience on light microscopy.

## Introduction

Malaria remains the most important parasitic disease as over one hundred countries worldwide are endemic [1]; the World Health Organization (WHO) estimates that malaria caused 197 million cases and 584 thousand deaths during 2013 [2]. Greece has been malaria-free since 1974 [3,4]. Until 1999, a number of imported cases have been reported each year and only sporadic autochthonous cases during the following decade [5]. During the 2011 and 2012 transmission seasons (May to November), outbreaks took place in an agricultural area (Evrotas municipality, Lakonia regional unit, South Greece), while sporadic locally acquired cases were recorded throughout the country. Forty two autochthonous cases were reported in 2011 and 20 autochthonous malaria *vivax* cases were recorded in 2012 [6]. The estimated incidence of malaria cases in Greece was extremely low in specific agricultural areas of the country where immigrants from endemic countries lived and worked. In particular, the cumulative incidence of autochthonous malaria cases in Evrotas municipality was 0.26% from 2011 to 2012. For the same period, the cumulative incidence of autochthonous malaria cases in other areas (e.g. Marathon municipality in East Attica region unit) was even lower than Evrotas and was estimated at 0.009%.

In January 2012, an “Integrated surveillance and control programme for West Nile virus and malaria in Greece” (MALWEST Project) was launched. Regarding malaria, the following actions were implemented: a) focus investigation in all autochthonous cases, b) an active case detection programme in areas with autochthonous cases, c) a mass screening program in immigrant and native populations in the Evrotas municipality and at points of entry. The main goal was to detect and treat malaria cases or carriers of the malaria parasite.

SD Bioline Malaria Ag Pf/Pan Rapid Diagnostic Test (RDT) that detects hrp-2 and pan-LDH antigens was introduced in the above interventions for malaria diagnosis, while laboratory confirmation of suspected malaria cases was obtained by the examination of blood specimen sent to the National Malarial Reference Center (National School of Public Health, NMRC). RDT is a lateral flow test that can detect malaria antigens in a small amount of blood (5 μL) and is based on the immunochromatographic principles—capture of parasite antigen using monoclonal antibodies against a malaria antigen [7]. It is estimated that over 200 different RDTs are commercially available [8], while a number of over 74 million RDTs were distributed during 2011, 72% of them in Africa, 22% in Southeast Asia and 4% in Eastern Mediterranean [9]. However, information is limited in regards to the evaluation of the use and performance of RDTs in non-endemic areas [8]. As Greece had been malaria-free for over 35 years, current experience on light microscopy regarding malaria diagnosis is quite low. Therefore, the effectiveness of RDT was assessed in order to find out whether this diagnostic tool could be routinely used for the diagnosis of malaria in a non-endemic country.

Conventional microscopic examination of both thick and thin Giemsa stained blood smears has been widely accepted as the examination of choice for malaria diagnosis [10,11,12], but since the quality of microscopy-based diagnosis is frequently compromised [13], PCR seems to have been gaining ground in accurate malaria diagnosis [14]. Thus, this study compares the field application of the RDT for malaria diagnosis against light microscopy and PCR.

## Materials and Methods

### Study population—Sample collection

Our study population included immigrants and Greek population tested for malaria with RTDs, microscopy and PCR during active case detection (visits for fever/symptoms screening every 15 days in immigrants’ quarters), focus investigation (investigation of all residents around the case’s house in a distance of 100 m) and mass screening activities that took place in regions where autochthonous cases of malaria occurred: Evrotas municipality (Lakonia regional unit, South Greece), where the outbreak took place, Marathon municipality (East Attica regional unit), Thebes municipality (Viotia regional unit, Central Greece) and Sofades municipality (Karditsa regional unit, Central Greece). RDT was performed by field health professionals on site. The health professionals were all medical doctors who received theoretical and practical training on RDTs organized by NMRC.

The blood samples were placed in EDTA tubes and transferred to the Peripheral Health Centres where the slides for light microscopy were prepared and tested. The slides were prepared and read by microbiologists who received detailed theoretical and practical training organized by NMRC and their performance was monitored by participation in external quality control scheme. The NMRC also participated successfully in external quality control scheme. Then both slides and EDTA tubes were transferred to the NMRC in iceboxes (0–5°C) within <6 hours for confirmation and PCR.

### Diagnostic tools

The diagnostic tools used were RDT, PCR and light microscopy (peripheral blood smears).

#### RDT

The SD Bioline Malaria Ag P.f./Pan 05FK60 rapid test was used in our study. The test was performed and the results interpreted according to manufacturer’s instructions.

In case the control line did not appear, the test was considered as invalid and it was repeated immediately. Test kits were stored at a dry place (25–28°C).

#### PCR

The first stage was the DNA extraction from 400 μL of fresh blood that was performed using either the Purelink Genomic DNA mini kit or the iPrep Purelink gDNA Blood kit with the iPrep Purification Instrument (Invitrogen, Carlsbad, CA) in a final extraction volume of 100 μL. PCR for malaria detection and discrimination between *P*. *falciparum* and *P*.*vivax* was performed using primers PL3, PL4 and PL5 [15] in a final reaction volume of 30 μL into which 3 μL of the extracted DNA was added. Amplification products were run on a 2% agarose gel and samples producing the 266 bp band were considered positive for *P*.*vivax*. Unlike other two-step procedures that are time-consuming and labor intensive, the present PCR is a single-step method (which is a very useful feature for routine laboratory practice) and also with very satisfactory detection levels [15].

#### Light microscopy

The smears were prepared from EDTA anticoagulated venous blood. To prepare the thick smear, a blood drop was put on the one edge of the slide and then spread in a circle with the use of another’s slide sharp corner. It was then dried for about half an hour and it was not fixed with menthanol. In this way, red blood cells were hemolyzed and thus the microscopist could see leucocytes and malaria parasites on the smear recognizing specific forms of parasite life cycle (e.g. trophozoites, gametocytes, schizonts) [16]. At least one hundred (100) good fields of a thick smear were carefully examined for about 10 minutes. After that a slide could be declared negative. If parasites were seen, further 100 fields were examined to identify a potential mixed infection. To prepare a thin film, a blood drop was spread on a slide and then dried for about 10 minutes and fixed in menthanol. In this case, at least four hundred (400) fields of a thin slide were examined for about 30 minutes. Species confirmation and parasite densities were obtained by examining the thin film. The result was expressed in a percentage of Red Blood Cells (RBC) parasites.

### Ethics approval

The field application of SD Bioline Malaria Ag Pf/Pan RDT in Greece was approved by the Committee for Vector Borne Diseases in the Hellenic Centre for Disease Control and Prevention (HCDCP) and by the Review Board of the Greek Ministry of Health. Written consent statement was completed by all participants. In case of children participated in the study, the written consent statement was signed by parents or guardians. Moreover, mediators/translators participated in all activities related to immigrants and supported the process of signing the written consent statements, which were translated in four languages.

### Statistical analysis

Results data on the three diagnostic tests for malaria (PCR, light microscopy and RDT) were entered in a predesigned specific database. SPSS version 17.0 software was used for the statistical analysis of the data. Sensitivity, specificity, PPV, negative predictive value (NPV) and positive and negative likelihood ratio (LR) were calculated for the above mentioned tests along with their 95% confidence intervals (95% C.I.). In order to be able to calculate the positive likelihood ratio, the value of 100% of specificity was replaced with 99.5%. The level of agreement between various diagnostic tests was assessed through the calculation of Kappa value.

## Results

**Fig 1:**
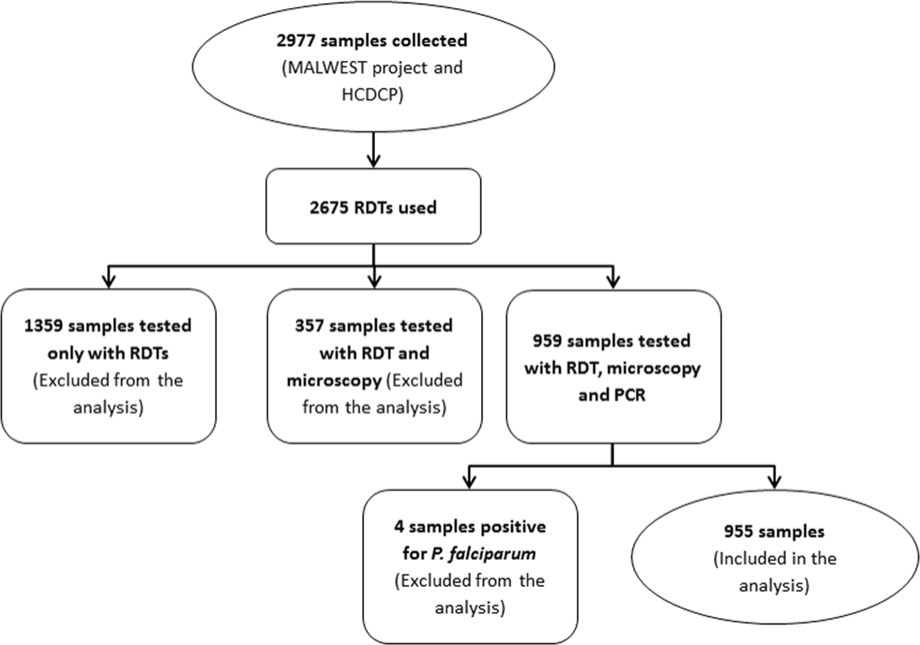
Flow chart of all samples collected for malaria in Greece (2012).

The total number of samples obtained during 2012 through the coordinate actions of MALWEST project and HCDCP is presented in Fig. 1. The analysis was restricted in those samples that were tested with all three diagnostic tools (PCR, light microscopy and RDT). In particular, out of 959 samples that were examined with all three diagnostic tools, four samples were positive for *P*. *falciparum* (all imported cases) in both microscopy and PCR and thus they were not included in the analysis (total number of 955).

As shown in Table 1, out of 955 samples included in the analysis, 46 were Greeks and 909 immigrants, the majority of whom from malaria endemic countries (e.g. Pakistan, Afghanistan). Sixteen of the immigrants were from Romania and Morocco (non-endemic countries); however, the immigrants from both endemic and non-endemic countries stayed in the same quarters in the agricultural regions where autochthonous cases occurred. The arrival date was recorded in only 635 out of 909 immigrants (mean duration of stay in Greece: 33.9 months) because most of the immigrants were undocumented and the information collected was self-reported. Of the 955 cases, 93 (9.7%) were tested with the three diagnostic tools in the context of symptoms compatible with malaria (including fever). These individuals were identified either through active case detection or through the Peripheral Health Centers or hospitals in the regions where autochthonous cases occurred. The remaining 862 samples were tested within the focus investigation and the mass screening activities. In particular, 210 (21.9%) were tested within the focus investigation and 652 (68.4%) within the mass screening activities.

**Table 1:** Description of samples included in the analysis.

Number of samples	Data for study population	Immigrants	Greek population
**955 included in the analysis**	Samples distribution	909	46
	Mean age	Mean age: 26.7 years old (range: 3–67)	Mean age: 27.0 years old (range: 1–91)
	Country of origin	Pakistan: 830/909 (91.3%), Afghanistan: 37/909 (4.1%), Bangladesh: 15/909 (1.7%), India: 11/909 (1.2%), Romania: 10/909 (1.0%), Morocco: 6/909 (0.7%)	Greece
	Duration of stay in Greece	Recorded for 635/909, mean duration of stay: 33.9 months (range: 1–264 months)	N/A
**93 samples tested in context of symptoms (fever)**	Samples distribution	75	18
	Mean age	Mean age: 26.5 years old (range: 10–49)	Mean age: 49.2 years old (range: 2.5–91)
	Country of origin	Pakistan: 68/75 (90.7%), Afghanistan: 4/75 (5.4%), Bangladesh: 1/75 (1.3%), Romania: 1/75 (1.3%), Morocco: 1/75 (1.3%)	Greece
	Duration of stay in Greece	Recorded for 36/75, mean duration of stay: 33.4 months (range: 3.5–60 months)	N/A
**45 PCR positive samples**	Samples distribution	34	11
	Mean age (45 PCR positive samples)	Mean age: 23.8 years old (range: 10–49)	Mean age: 50.5 years old (range: 23–82)
	Country of origin	Pakistan: 21/34 (61.8%), Afghanistan: 9/34 (26.5%), Bangladesh: 2/34 (5.9%), Romania: 1/34 (2.9%), Morocco: 1/34 (2.9%)	Greece
	Duration of stay in Greece	Recorded for 31/34, mean duration of stay: 32.4 months (range: 5–60 months)	N/A

Out of 45 PCR positive samples, 11 were Greeks and 34 immigrants. The main demographic data of these samples are given in Table 1. Out of 45 positive PCR malaria cases, 19 had symptoms compatible with malaria (including fever) and only 21 of them (46.7%) were hospitalized. The most common symptoms were headache (46.7%), fever >38°C (42.2%), myalgia and arthralgia (31.1%), vomiting or nausea (20%) and chills (17.7%), whereas anemia was recorded in 24.4%. According to the HCDCP guidelines, a combination of chloroquine for two days and primaquine for 14 days was the antimalarial regimen used to treat all 45 malaria cases.

Table 2 illustrates the results of RDT for *P*. *vivax* in comparison to PCR as the reference diagnostic method. The sensitivity and specificity of RDT against PCR was 95.6% (95% C.I.: 84.8–99.3) and 100% (95% C.I.: 99.6–100.0), respectively. The PPV was 100% (95% C.I.: 91.7–100.0), while the NPV was 99.8% (95% C.I.: 99.2–100.0). The positive likelihood ratio (LR+) was 191.1, while the negative likelihood ratio (LR-) was 0.044. Kappa (K) value was 0.976 (95% C.I.: 0.943–1.000). Out of two samples that were RDT negative and PCR positive, one was found positive and one negative when examined with light microscopy. The RDT negative and light microscopy positive was taken from a lady of Greek origin with mild symptoms compatible with malaria, while the RDT negative and the light microscopy negative was obtained from an immigrant from malaria endemic country who had a severe clinical picture (high fever, myalgia, headache, symptoms from the respiratory and the gastrointestinal system).

**Table 2:** RDT and PCR results of samples for *P*. *vivax*.

		PCR		
		Positive	Negative	Total
**RDT**	**Positive**	43	0	43
	**Negative**	2	910	912
	**Total**	45	910	955

In Table 3, the results of light microscopy in comparison to PCR are presented. The sensitivity and specificity of microscopy against PCR was 84.4% (95% C.I.: 70.5–93.5) and 100% (95% C.I.: 99.6–100.0), respectively. The PPV was 100% (95% C.I.: 90.7–100.0), while the NPV was 99.2% (95% C.I.: 98.4–99.7). The LR+ was 168.9, while the LR- was 0.156. K value was 0.912 (95% C.I.: 0.847–0.977). Out of the seven samples that were found to be PCR positive but microscopy negative, six were RDT positive, while the seventh one was also negative when tested with the RDT.

**Table 3:** Microscopy and PCR results of samples for *P*. *vivax*.

		PCR		
		Positive	Negative	Total
**Microscopy**	**Positive**	38	0	38
	**Negative**	7	910	917
	**Total**	45	910	955

Table 4 depicts the results of RDT for *Plasmodium vivax* in comparison to light microscopy. The sensitivity and specificity of RDT against microscopic examination was 97.3% (95% C.I.: 86.1–99.6) and 99.4% (95% C.I.: 98.6–99.8) respectively. The PPV was 86.1% (95% C.I.: 72.1–94.7), while the NPV was 99.9% (95% C.I.: 99.4–100.0). The LR+ was 148.8, while the LR- was 0.027. K value was 0.910 (95% C.I.: 0.843–0.976). It is worth mentioning that the sample which was RDT negative and microscopy positive was further confirmed by a positive PCR result. The level of parasitemia in the sample tested RDT negative and light microscopy positive was estimated at 0.04%. In addition, the six samples that were RDT positive but microscopy negative were found also positive with PCR.

**Table 4:** RDT and microscopy results for *P*. *vivax*.

		Microscopy		
		Positive	Negative	Total
**RDT**	**Positive**	37	6	43
	**Negative**	1	911	912
	**Total**	38	917	955

Moreover, analysis was conducted to compare the performance of RDT against PCR as the reference diagnostic method in 93 symptomatic individuals and 862 samples collected during focus investigation and mass screening activities. Regarding 93 symptomatic individuals, the sensitivity and specificity of RDT against PCR was 94.7% (95% C.I.: 71.9–99.7) and 100% (95% C.I.: 93.9–100.0). The PPV was 100% (95% C.I.: 78.1–100.0), while the NPV was 98.7 (95% C.I.: 91.8–99.9). The LR+ was 189.5, while the LR- was 0.052. K value was 0.966 (95% C.I.: 0.901–1.000). Regarding the remaining 862 samples, the sensitivity and specificity of RDT against PCR was 96.2% (95% C.I.: 78.4–99.8) and 100% (95% C.I.: 99.4–100.0). The PPV was 100% (95% C.I.: 83.4–100.0), while the NPV was 99.9% (95% C.I.: 99.2–100.0). The LR+ was 193.5, while the LR- was 0.039. K value was 0.979 (95% C.I.: 0.940–1.000).

## Discussion

Our results suggest that RDTs demonstrated high sensitivity, specificity, and positive predictive value for the diagnosis of *P*. *vivax* malaria in a non-endemic country. The sensitivity of the used RDT test against PCR and microscopy was 95.6% and 97.4%, respectively, while the specificity was 100% and 99.4%, respectively, accompanied by high PPV especially when comparing RDT performance to the gold standard PCR. The RDT seemed to have similar performance both in symptomatic individuals and individuals participated in focus investigation and mass screening. In addition, the largest agreement among diagnostic tools was the one between RDT and PCR, indicating the good performance of RDT even when used in non-endemic country.

Previous studies usually followed a comparison of RDT performance to microscopic and/or PCR results. In Belgium, which is a non-endemic country, the evaluation of SD Bioline Malaria Ag Pf/Pan revealed a sensitivity of 92.9% [17]. On the other hand, a number of surveys regarding the evaluation of SD Bioline Malaria Ag Pf/Pan test against microscopy took place in endemic countries (Colombia, Korea, Myanmar). In Colombia, sensitivity, specificity, PPV and NPV for SD Bioline Pf/Pv test were estimated at 92%, 98.7%, 94.5% and 98.1%, respectively [18]. In Korea, sensitivity of RDT for *P*. *vivax* was ranged from 86.8% to 92.7% [19,20], while in Myanmar, sensitivity, specificity, PPV and NPV for P. vivax/malariae was estimated at 79.4%, 98.5%, 98.7%, 96.3% and 91.6%, respectively [21].

In our study, there were two false negative RDTs, while PCR was positive. One of these cases had severe symptoms and the possible cause of false negative in both RDT and light microscopy was that blood sample was taken beyond the period of fever paroxysms. This explanation could be the most acceptable scenario, as two days later all three diagnostic tools were used in a new blood sample came up positive. The second case was a Greek lady with mild symptoms who had negative RDT and positive light microscopy and PCR. It should be noted that, according to our NMRC, the level of parasitemia was extremely low (0.04%) and beyond the detection limit of RDT (~2000 parasites/μL).

Currently, the WHO recommends confirmation of a malaria suspected case before treatment starts, either by RDT or by microscopic examination. The choice of the diagnostic tool depends on the circumstances met in each setting; it has mostly to do with technical skills and epidemiological facts. The presence of high or low prevalence of the disease is related to the PPV of the diagnostic tool, while it is already mentioned how the lack of expertise is directly connected to the conventional light microscopy efficiency. Additionally, when high-skilled light microscopy is not available, quality-assured RDT are considered acceptable for malaria diagnosis [22,23].

Light microscopy can be sensitive and specific diagnostic tool [24] when used by a well-trained microscopist, giving the advantages of mixed infections identification, parasitemia level determination, treatment success monitoring, requiring little laboratory equipment [12]. However, false positive results may occur due to wrong smear preparation (artifacts that may be considered as parasites) or normal blood components that may confuse the microscopist, while false negative results may appear due to small number of examined fields [7]. Microscopic examination may not be able to detect low parasitemias and is a method whose value and effectiveness is highly based on the competency of the microscopist [12]; in a non-endemic country it seems quite reasonable that the microscopists, if not working in parasitological departments or reference centers or not having had experience from an endemic region, are not familiar with malaria microscopy features. Although training courses (e-learning and face-to-face) for microbiologists and laboratory technicians by malaria experts of the NMRC were organized in the context of the MALWEST project it was not expected that all malaria cases could be easily diagnosed and especially those occurring in semi-immuned immigrants. Thus, it is rather expected for a non-malaria setting to display weaknesses in microscopic diagnosis of the disease [25].

Molecular testing with PCR seems to have the highest levels of sensitivity, detecting even one parasite per μL of blood [15] and subpatent infections, and also identifies the causative species [26]. It can be used for microscopy or RDT confirmation and may be the only safe choice for low parasitemia detection and for finding asymptomatic infections in non-endemic settings [27], however it could be considered rather expensive.

Both in endemic and non-endemic areas, RDTs are increasingly being used as a malaria diagnostic tool [28, 29]. The main advantages are that it is a simple and cheap method, it does not require special equipment or intensive training, it is a very quick method providing results in only 15–30 minutes and its interpretation is more objective than that of light microscopy [30]. RDTs can diagnose malaria in settings where the distance from effective microscopy services is an inevitable fact [30] and also during disease surveillance or outbreak investigations [31]. On the other hand, it is not a qualitative method as it cannot measure the parasitemia levels and thus the infection severity and may not be able to detect very low parasitemias/asymptomatic infections [30]. We chose the SD Bioline Malaria Ag P.f./Pan 05FK60 test, as it was proved to be one of the best performers in the WHO/TDR/FIND/CDC evaluation programme [32], despite one of its shortcomings was that it cannot differentiate specifically for *P*. *vivax*. It has been suggested that due to its drawbacks, the RDT could not be probably established as the unique diagnostic method in non-endemic areas, where conventional microscopic examination is required to confirm the diagnosis, as the RDT may not detect a low parasitemia infection [33], in comparison to microscopy which is able to detect lower levels of parasitemia than RDT. The majority of RDT tests show high level of plasmodium detection at parasitemia of at least 2000 parasites/μL [34]. Similar detection limit was identified in our study, as the level of parasitemia of light microscopy positive and PCR negative sample was estimated at 0.04% (~500 parasites/μL). Only a few studies concerning RDT evaluation have taken place in non-endemic settings, where, in addition, only a small number of RDTs was used [8]. Even though previous evaluations support that the microscopy cannot be surpassed by RDT use, our results indicate that RDTs perform equally and sometimes even better when compared to conventional microscopy and can be used in control activities decreasing the time between diagnosis and treatment.

Our study has some limitations which should be taken into account. The main limitation of our study is the small number of 45 PCR positive samples which affected the confidence interval (C.I.) of the RDT sensitivity assessment, while the C.I. of the specificity assessment was narrower due to higher number of samples. Moreover, this study was not a clinical trial (Phase III) but a descriptive observational study and as such has the limitations and the biases (possible selection bias) of an observational study. Thus, the results could not be directly compared to those of trials which evaluated the performance of RDTs [35,36,37].

In conclusion, our field study suggested that the RDT used for malaria diagnosis in a non-endemic area demonstrated satisfactory agreement with traditional tools of malaria diagnosis (light microscopy, PCR).
